# Comparative Dynamics and Distribution of Influenza Drug Resistance Acquisition to Protein M2 and Neuraminidase Inhibitors

**DOI:** 10.1093/molbev/mst204

**Published:** 2013-11-07

**Authors:** Vanessa Garcia, Stéphane Aris-Brosou

**Affiliations:** ^1^Department of Biology, University of Ottawa, Ottawa, Ontario, Canada; ^2^Department of Mathematics and Statistics, University of Ottawa, Ottawa, Ontario, Canada

**Keywords:** Adamantane, Oseltamivir, influenza, drug resistance, molecular dating, phylogenetics

## Abstract

Although efficient influenza vaccines are designed on a regular basis, the only protection of human populations against an unforeseen virus such as during the H1N1 pandemic in 2009 might be antiviral drugs. Adamantanes and neuraminidase inhibitors (Oseltamivir) represent two classes of such drugs that target the viral matrix protein 2 and neuraminidase, respectively. Although the emergence of resistance to both drugs has been described, the timing and spread of the acquisition of either single or dual resistances by different hosts is still unclear. Using a multilayered phylogenetic approach based on relaxed molecular clocks and large-scale maximum likelihood approaches, we show that Adamantane resistance evolved multiple times in various subtypes and hosts, possibly in breeding contexts (swine); and Oseltamivir resistance was also found in different subtypes and hosts, but its transmission is only sustained in humans. Furthermore, the dynamics of the emergence of antiviral resistance were examined for each drug. This showed that although the first mutations conferring resistance to Adamantanes precede US Food and Drug Administration (FDA) approval, general resistance emerged 15–38 years post-drug approval. This is in contrast to Oseltamivir resistance mutations that emerged at most 7 years after FDA approval of the drug. This study demonstrates the power of large-scale analyses to uncover and monitor the emergence dynamics of drug resistance.



## Introduction

Worldwide surveillance has allowed us to monitor the emergence of new strains of influenza A viruses and to produce vaccines based on the projected dominant strains. However, as in the case of the 2009 H1N1 pandemic, the emergence of a new strain can be unforeseen and leave health authorities little to no time to develop an effective vaccine to immunize human populations. In such cases as well as with immunocompromised patients, antiviral drugs are critical as therapeutic or prophylactic agents (Maugh 1979) against influenza (Fitzgerald 2009). To this effect, two broad classes of drugs, matrix protein 2 (M2) and neuraminidase (NA) inhibitors (NAIs), have been approved and used in the treatment of influenza (Cheng et al. 2009; Salter et al. 2011).

The class of drugs known as Adamantane was approved by the US Food and Drug Administration (FDA) in 1966 (Maugh 1979) and is only effective against influenza A strains. The Adamantanes are comprised of amantadine and rimantadine, which target the M2 protein of the virus. The M2 protein functions as an acid-activated ion channel and is required for the release of the nucleoprotein following its fusion with the endosomal membrane (Hay et al. 1986; Bright et al. 2006). Adamantanes inhibit viral replication by preventing M2 channel opening, thus interfering with viral uncoating during endocytosis (Hay et al. 1986; Salter et al. 2011).

The second class of anti-influenza drugs, NAIs, were FDA approved in 1999 (Gubareva et al. 2000) and include Oseltamivir (Tamiflu, by Roche) and Zanamivir (Relenza, by GlaxoSmithKline). NAIs target the NA protein of influenza which is responsible for the cleavage of host cell sialic acid residues allowing the release of the budding virions (Tashiro et al. 2009). NA is also important in the establishment of upper respiratory infections, as cleavage of sialic acid on mucosal surfaces exposes the epithelial cells to the virus. NAIs prevent the release of virions from an infected cell and thereby reduce both the upper respiratory infections and the duration of symptoms (Lackenby, Hungnes, et al. 2008; Tashiro et al. 2009).

The widespread use of Adamantanes and NAIs against influenza has led to the emergence of resistant virus strains. Adamantane resistance is characterized by a mutation in one of five sites (at positions 26, 27, 20, 31, or 34) in the M2 gene, though the most commonly observed mutation is the S31N (Belshe et al. 1988; Wang et al. 2013). Each mutation results in either reduced binding of the drugs to their M2 ligand or in the expansion of the M2 channel, both of which allow the channel to exert its function in the presence of the drug. NAI resistance occurs through mutations of the viral NA gene that, much like the M2 mutations, reduce the affinity of the drugs for its ligand (Lackenby, Hungnes, et al. 2008; Lackenby, Democratis, et al. 2008). The most common mutation in NA is H274Y, although other mutations such as E119V, N294S, and R292K have been described (Sheu et al. 2011). In addition to “single-resistant” influenza phenotypes, “dual-resistant” strains, which are resistant to both M2 inhibitors and NAIs, have recently been detected (Sheu et al. 2008, 2011). The spread of dual-resistant virus strains is concerning as no approved anti-influenza drugs are effective in treating patients.

Tracing the evolution of drug resistance mutations allows for the identification of resistant strains and gives a timeline to the spread of resistance since their approval by the FDA. Though previous studies have focused on the emergence of resistance to a particular drug in a particular influenza subtype, such as Adamantane in H3N2 viruses circulating in humans (Simonsen et al. 2007) or over all subtypes circulating in European swine (Krumbholz et al. 2009), very few studies have focused on resistance to both classes of drugs across all possible subtypes and hosts (Sheu et al. 2011). Here, we unravel the dynamics of the emergence of resistance to both classes of drugs, starting from H1N1 viruses circulating worldwide since 1918, before casting the results in the very general context of the emergence of these resistances across all influenza A sequences publicly available as of July 2013. Our results not only show striking similarities in the evolution of both types of antiviral resistance but also highlight some critical differences in the timing of their emergence.

## Results and Discussion

### Oseltamivir Resistance Quickly Emerged After FDA Approval

**Fig. 1.**
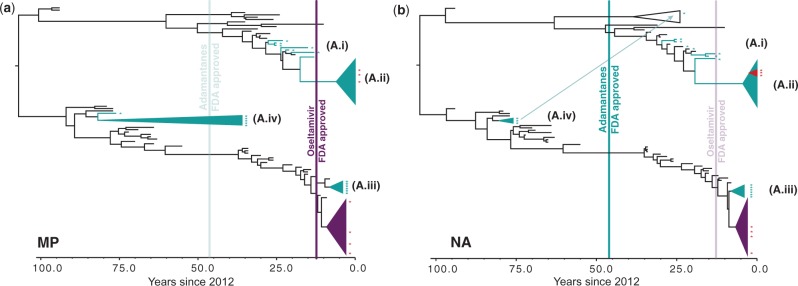
Dated phylogenies of drug-resistant influenza A/H1N1 gene segments: (*a*) for the MP gene and (*b*) for the NA gene. Black branches indicate an absence of Adamantane or Oseltamivir resistance, whereas blue branches and/or asterisks (*) indicate the presence of Adamantane resistance, purple the presence of Oseltamivir resistance, and red both Adamantane and Oseltamivir resistance. The horizontal axis represents time in years since 2012, whereas the vertical bars show FDA approval for Adamantane (blue; 1966) and Oseltamivir (purple; 1999); colors of these bars are shaded for gene segments not involved in a particular resistance. Identities of Adamantane resistance clades are shown between parentheses. An arrow represents the main reassortment event (in NA). Node posterior probabilities are shown in supplementary figure S1, Supplementary Material online.

The Bayesian relaxed molecular clock analysis of our small targeted analysis of H1N1 complete genomes collected worldwide suggests that single Oseltamivir drug resistance arose only once in our sequence catchment, as shown by the presence of a single clade conserved among seasonal virus segments containing all Oseltamivir-resistant sequences (fig. 1*a* and supplementary fig. S1, Supplementary Material online; purple clade). Three observations can be made from this H1N1-targeted analysis: in all the retrieved sequences, single-drug resistance to Oseltamivir is 1) conferred by the H274Y mutation in NA, 2) limited to human hosts, and 3) limited to seasonal (prepandemic) H1N1 viruses, while being highly prevalent in this latter group. These results are consistent with previous observations on the emergence of this drug resistance between 2008 and 2009 (Dharan et al. 2009; Meijer et al. 2009).

Two factors can explain this pattern. First, acquisition of single-drug resistance by patients under treatment is low, with rates of 1–2% in adults (Jackson et al. 2000) and 5–6% in children (Whitley et al. 2001). Second, since the approval of the drug by the FDA back in 1999, there may not yet have been sufficient time as of early 2013 for the independent acquisition of mutations conferring single-drug resistance to Oseltamivir or for reassortment into swine and/or avian H1N1 strains. Figure 1 and supplementary figure S1, Supplementary Material online, also confirm the recent and sporadic emergence of Oseltamivir resistance in the context of pandemic H1N1/2009 viruses that are already Adamantane resistant (Hurt et al. 2009). These latter mutations are all H274Y, and the phylogenetic analysis suggests that they are all derived from a unique mutation with a high posterior probability (*P* = 0.98; supplementary fig. S1, Supplementary Material online) in the ancestor of A/Bethesda/NIH106-D14/2009 and A/Boston/678/2009, divergence which occurred between 2008 and 2009 (fig. 1*a*). Phylogenetic evidence suggests that these pandemic viruses carrying the de novo H274Y mutation reassorted repeatedly with other pandemic H1N1 viruses circulating in humans (fig. 1 and supplementary fig. S1, Supplementary Material online), a process that may favor the spread of this resistance in N1 viruses but that, as of early 2013, is still limited to H1N1/2009.

**Fig. 2.**
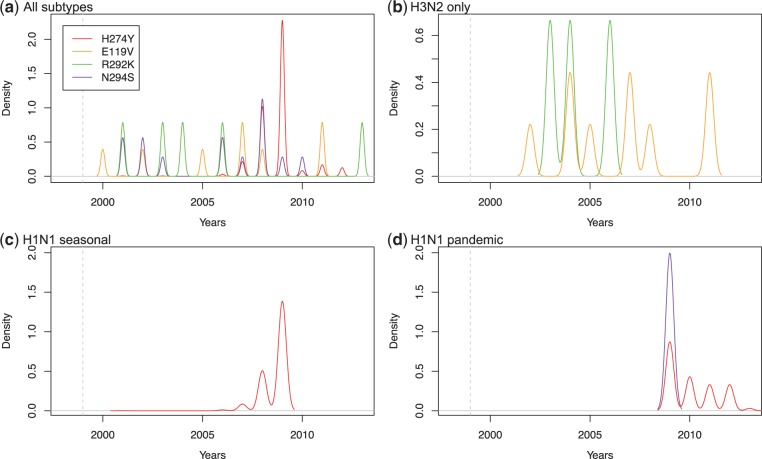
Dynamics of the presence of the mutations conferring resistance to Oseltamivir. The four known mutations are mapped through our extended data set of 19,932 NA sequences: H274Y (red), E119V (orange), R292K (green), and N294S (purple). Presence (in IVR) densities are represented: (*a*) across all subtypes and all hosts, (*b*) in H3N2, (*c*) in seasonal H1N1 viruses, and (*d*) in pandemic viruses. The dashed gray vertical line represents the date of approval of Oseltamivir by the FDA (1999).

An exhaustive survey across the Influenza Virus Resource (IVR) database (20,888 sequences—see Materials and Methods) of the four mutations known to be involved in Oseltamivir resistance (H274Y, E119V, N294S, and R292K) reveals a number of critical points in terms of the distribution, timing, and emergence of these mutations. First, we note that H274Y is the most common mutation (fig. 2*a* and supplementary table S5, Supplementary Material online) and seems to be exclusively limited to the N1 context in both seasonal (fig. 2*c*) and pandemic strains (fig. 2*d*) (Hurt et al. 2009) and human hosts (supplementary table S5, Supplementary Material online: only one avian case—out of 648 H274Y mutations). However, a very small number of N294S mutations are also found in pandemic H1N1 viruses (fig. 2*d*); more generally, all four mutations exist in the N1 context (supplementary table S2, Supplementary Material online). Moreover, although H3N2 is dominated by the E119V and R292K mutations, H274Y is never identified here in the N2 context (fig. 2*c*).

**Fig. 3.**
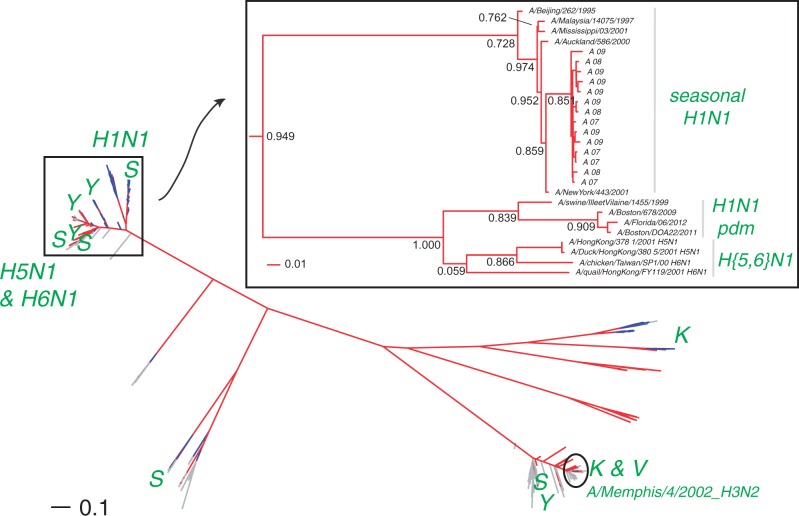
Phylogenetic distribution of the mutations conferring resistance to Oseltamivir in our extended data set of 20,888 NA sequences. Subtypes are color-coded: H1N1 in red, H3N2 in blue, and others in gray. Mutations are single-letter coded: Y for H274Y, V for E119V, K for R292K, and S for N294S. Key sequence names are shown. The inset is a magnification of the subtree containing H1N1, H5N1, and H6N1 resistant sequences (other sequences omitted for clarity). Resistant sequences included in the small data set (fig. 1) are coded as “A” followed by the last two digits of their collection year. Additional sequences are also indicated to show the origin of early non-H274Y resistance. Scale bars are in expected number of substitution per site.

Second, figure 2*a* and supplementary table S2, Supplementary Material online, show that the first resistance mutations found in human hosts appeared in 2001 (H274Y: A/Mississippi/03/2001_H1N1) and in 2002 (E119V: A/Memphis/4/2002_H3N2) despite low usage of Oseltamivir (<2 million doses; Hurt et al. 2009). Yet, even in this low-use situation, the same mutations can be found in other genetic/host contexts, earlier: E119V in 2000 (A/chicken/Taiwan/SP1/00_H6N1); N294S in 2001 (both in a duck A/Duck/Hong Kong/380_5/2001_H5N1 and in a human A/Hong Kong/378_1/2001_H5N1); and R292K in 2001 (A/quail/Hong Kong/FY119/2001_H6N1; fig. 2 and supplementary table S4, Supplementary Material online). Although mutation N294S has previously been reported in H5N1 viruses (Le et al. 2005; Yen et al. 2007), mutations in H11N2 or H5N5 (supplementary table S2, Supplementary Material online) had not previously been found. The phylogenetic analysis of this extended NA data set (fig. 3) shows that the mutation in A/Mississippi/03/2001_H1N1 is most likely a sporadic event that did not propagate as its placement on the tree is “between” two sensitive strains with node support values >0.72 (fig. 3, see inset). The mutations in H5N1 were most likely linked to the 1996–2004 avian flu episodes in South East Asia (Hill et al. 2009) and, just as the mutations in H6N1, are not related to the mutation found in H1N1 pandemic viruses. Only 12 H3N2 viruses, all circulating in humans, were found to be potentially resistant to Oseltamivir (supplementary table S2, Supplementary Material online); although this low number may reflect the poor protective effect of non-H274Y mutations (Yen et al. 2005), the reason why H274Y is not found in H3N2 may be due to 3D constrains, but it is still unknown. Finally, the repeated and independent origin of all mutations, except maybe E119V in N2 contexts (fig. 3), may be linked to the reduced fitness of this particular mutation in reverse genetics experiments compared with all other resistance mutations (Hayden and de Jong 2011—albeit compensatory mutations may exist elsewhere in the genome of actual viruses).

### Adamantane Resistance Evolved Multiple Times, Before FDA Approval of Adamantane

**Fig. 4.**
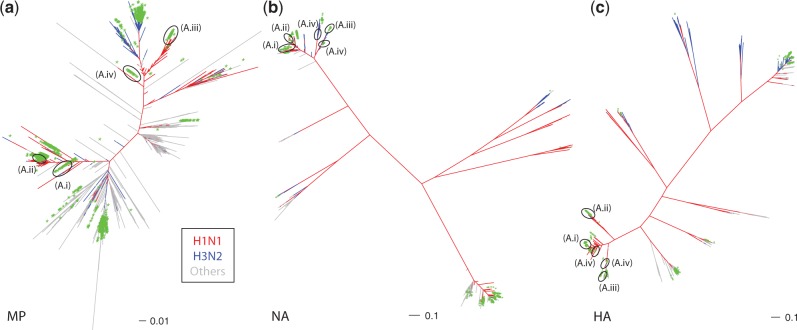
Phylogenetic distribution of the S31N mutation conferring resistance to Adamantanes in our extended data sets: (*a*) for the MP gene segment, (*b*) for NA (same tree as fig. 3), and (*c*) for HA. Sequences harboring the S31N mutation in MP have their phylogenetic placement indicated by an asterisk (*). The clades identified in the H1N1-targetted analysis (fig. 1*h*) are shown (A.i–A.iv). Subtypes are color-coded: H1N1 in red, H3N2 in blue, and others in gray. Scale bars are in expected numbers of substitution per site.

The phylogenetic trees generated for all gene segments in the H1N1-targeted analysis confirm that Adamantane resistance is more easily acquired than Oseltamivir resistance (fig. 1 and supplementary fig. S1, Supplementary Material online; Hurt et al. 2009). Instead of just one main clade of Oseltamivir resistance, viruses solely resistant to Adamantane form four distinct clades in our genome catchment (fig. 1*b*): (A.i) a swine clade comprising viruses collected between 1987 and 1999 and that diversified between 1980 and 1984, (A.ii) a clade containing human H1N1/2009 pandemic sequences that emerged in 2009 (Lemey et al. 2009; Smith et al. 2009), (A.iii) a clade comprising seasonal H1N1 strains collected between 2006 and 2008 and that diversified between 2005 and 2006, and (A.iv) a clade of Adamantane-resistant viruses sampled as early as 1934 and up to 1976, inferred to have diversified around 1927–1930 and that includes two strains of swine origin. Note that (A.iii) was only present in our sequence catchment for segments HA (hemagglutinin), NA, and MP (matrix protein) (supplementary fig. S1, Supplementary Material online) but may be more widely distributed. All the other clades are found to be present without evidence for reassortment across gene segments, with the exception of A/New Jersey/1976, which clusters within viruses circulating in North American swine in the case of PB1 or Eurasian swine for HA and NA. In the case of PB1 and NA, this reassortment/host change event can be dated back to 1964–1972 (PB1) and 1974–1975 (NA), whereas the uncertainty for HA is much larger (1945–1972) due to a long branch reflecting unsampled history. These results on reassortment are confirmed by the phylogenetic analyses of our extended data sets, which contain all available MP (*n* = 19,932 sequences), NA (*n* = 20,888), and HA (*n* = 24,168) segments in the IVR database (fig. 4). Surprisingly, these large-scale analyses are extremely well supported (supplementary fig. S2, Supplementary Material online; number of nodes with approximate likelihood-ratio test (aLRT) support values >70%, which corresponds to a 95% probability for the node to be correct [Hillis and Bull James 1993]: 87.70% for HA, 85.33% for NA, and 88.98% for MP) and show that apart from A/New Jersey/1976, reassortment does not seem to play a key role in the spread of single-drug resistance mutations in H1N1 viruses: the same four Adamantane resistance clades identified in the MP tree (fig. 4*a*) are also found in the NA tree (fig. 4*b*), whereas these clusters are “broken” in a segment (HA) that is not involved in drug resistance (fig. 4*c*).

Even if several mutations are known to confer resistance to Adamantane (Shiraishi et al. 2003), almost all the Adamantane single-drug resistant genomes sampled contain the S31N mutation, consistent with previous observations in H3N2 viruses (Simonsen et al. 2007; Nelson et al. 2009); here, only A/hvPR8/34 harbors a second mutation, V27A, in addition to the S31N mutation (supplementary table S2, Supplementary Material online). In our extended data sets, a search for these mutations reveals the existence of 60 (out of 19,932 MP sequences) rare viruses carrying dual mutations (supplementary table S3, Supplementary Material online). No MP segments were found with more than two mutations. Dual mutations are not restricted to H3N2 and H1N1 viruses or even to human hosts (supplementary table S3, Supplementary Material online) and, similar to previous reports (Hayden and de Jong 2011), no evidence of reassortment between the MP segment of Oseltamivir-resistant viruses and pandemic H1N1 viruses could be found (figs. 1 and 4). This contrasts with the finding that intra-subtype reassortment can lead to dual resistance, in this case under pressure of Adamantane overuse (Zhou et al. 2011). Our results suggest that monitoring and regulation of drug usage may be beneficial in the prevention of reassortment leading to dual-resistant phenotypes.

The dynamics of the acquisition of these mutations confirms the phylogenetic results shown earlier, that Adamantane resistance (mutations) emerged multiple times. Figure 1*b* suggests three to four independent acquisitions in H1N1 viruses, one of them in swine (clade (A.iv)). Figure 4*a* demonstrates that across all subtypes and all hosts present in the IVR database, Adamantane resistance evolved repeatedly a large number of times and independently of the subtype structure (fig. 4*b* and *c*) or of the host (supplementary table S3, Supplementary Material online). This result is consistent with clinical observation of rapid acquisition of Adamantane resistance in patients under treatment (Shiraishi et al. 2003), with the emergence of the S31N mutation even in absence of drug selective pressure (Simonsen et al. 2007), an observation consistent with the absence of any fitness effects of this mutation in reverse-genetics experiments (Abed et al. 2005).

Lending further support to the multiple acquisitions of Adamantane resistance is the presence of double-resistant sequences in the Oseltamivir clade (fig. 1 and supplementary fig. S1, Supplementary Material online; purple clade). Viruses in this clade all carry the same Oseltamivir resistance mutation on the NA gene segment (H274Y) but differ in their Adamantane resistance mutation: V27A (A/Texas/38/2009 and A/Kentucky/08/2009), S31N (A/West Virginia/02/2009), and G34E (A/Boston/72/2009). This suggests an initial acquisition of the H274Y mutation in all the double-resistant NA genes, followed by the emergence of many independent MP mutations.

**Fig. 5.**
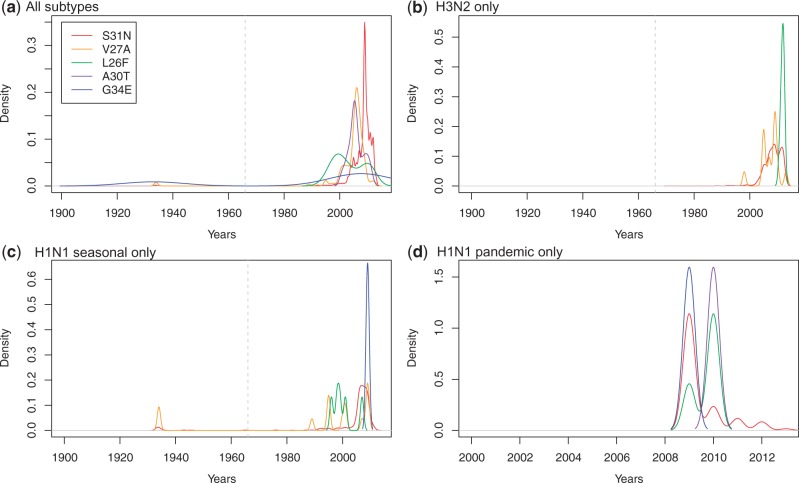
Dynamics of the presence of the mutations conferring resistance to Adamantane. The five known mutations are mapped through our extended data set of 24,168 MP sequences: S31N (red), V27A (orange), L26F (green), A30T (purple), and G34E (blue). Presence densities are represented: (*a*) across all subtypes and all hosts, (*b*) in H3N2, (*c*) in seasonal H1N1 viruses, and (*d*) in pandemic viruses (note the change of scale on the *x* axis for this panel). The dashed gray vertical line represents the date of approval of Adamantane by the FDA (1966).

One puzzling result observed both in the H1N1-targeted Bayesian analysis (fig. 1 and supplementary fig. S1, Supplementary Material online) and in the mutation analysis of the extended data sets is the presence of Adamantane resistance well before the FDA approval of Adamantane or even before the first report of antiviral activity back in 1963 (see Maugh 1979). When the yearly distribution of resistance mutations is plotted over all subtypes and hosts (fig. 5*a*), it is clear that three mutations (S31N, V27A, and G34E) were present in the early 1930s. Early S31N is found not only in humans but also in a number of birds (supplementary table S3, Supplementary Material online), mostly in seasonal H1N1 (fig. 5*c*), as well as in H5N3 and H7N7 (supplementary table S3, Supplementary Material online), but never in H3N2 (fig. 5*b*). In light of previous studies alone (Simonsen et al. 2007), this pattern could be interpreted as recurrent mutations whose fate is governed by drift and/or hitchhiking, as S31N alone does not seem to entail any fitness costs (Bean et al. 1989). However, S31N is not the only mutation circulating during the early 1930s in humans, as both V27A and G34E are found, all of them in H1N1 viruses. Even if all three mutations are neutral (Simonsen et al. 2007), it is difficult to explain why they would all appear within the same narrow time window 1933–1934 in H1N1 viruses circulating in humans without invoking either epistatic interactions among these mutations (hitchhiking) and/or early uncontrolled trials of Adamantane precursors (coincidently, Adamantane was indeed discovered in 1933: Landa et al. 1933).

### Comparative Dating of Resistance Emergence

**Fig. 6.**
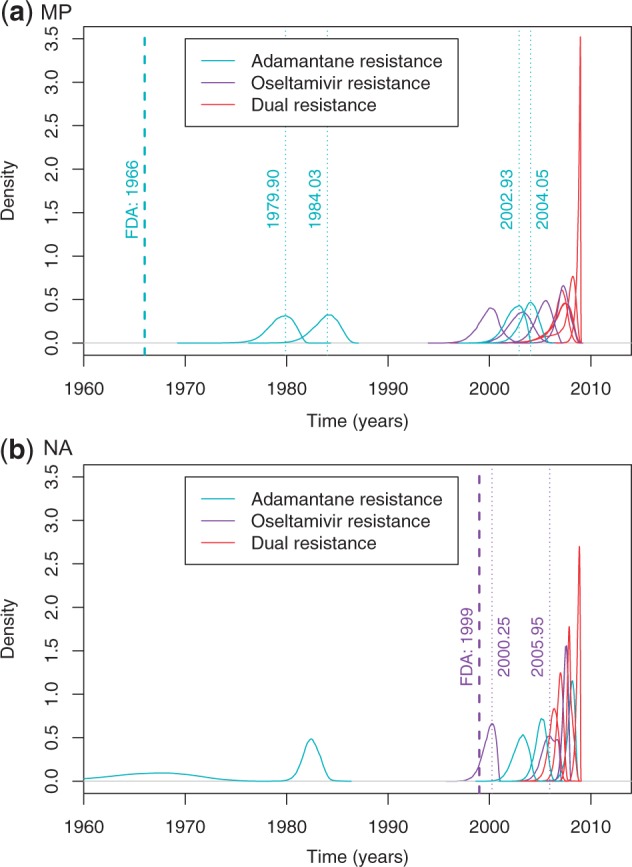
Dating the emergence of the mutations conferring resistance to Adamantane, Oseltamivir, and dual resistance under relaxed molecular clock models. (*a*) Posterior densities of dates estimated for the MP gene, responsible for Adamantane resistance. (*b*) Posterior densities of dates estimated for the NA gene, responsible for Oseltamivir resistance. In each case, the branch along which resistance emerged was mapped by parsimony, so that two dates are represented for each mutation: one at the beginning of the branch (the earliest date of appearance) and one at the end of the branch (the oldest date of appearance). The dashed vertical lines represent the date of approval by the FDA of Adamantane (*a*: 1966) and Oseltamivir (*b*: 1999).

Although the results above support clinical observations (Whitley et al. 2001) that Adamantane resistance evolves more easily than Oseltamivir resistance, most likely in the absence of resistance cost (Simonsen et al. 2007), our H1N1-targeted analysis (fig. 1 and supplementary fig. S1, Supplementary Material online) allows us to compare the earliest dates of emergence of these phenotypes after FDA approval of these drugs. If clade (A.iv) from the early 1930s is disregarded, Adamantane resistance can be estimated to have emerged between late 1979 and early 1984 (fig. 6*a*; base of clade (A.i)), that is, 14 years at the earliest after FDA approval of the drug. Note that this date is an underestimate of the lapse of time between FDA approval and emergence in the human population, as clade (A.i) circulated mostly in swine, possibly in a farming context (fig. 1 and supplementary fig. S1, Supplementary Material online) (Guan et al. 2004; Li et al. 2004, but see Krumbholz et al. 2009). Clade (A.iii) would date this most recent common ancestor between 2003 and 2004 (fig. 6*a*), in line with observations of the earliest sequences collected in early 2005 for H3N2 viruses (Simonsen et al. 2007).

Emergence of Oseltamivir resistance, on the other hand, presents a completely different dynamic: approved in 1999, the earliest date of appearance of H274Y is just a year later, while the oldest possible date is 7 years later (fig. 6*b*). Thus, although Oseltamivir resistance is more difficult to observe in individual patients under treatment, at the population level, Oseltamivir resistance spread faster than Adamantane resistance. This paradox may merely reflect differences in drug use patterns that may have intensified Oseltamivir use around 2005 when Adamantane efficacy plummeted, but this paradox should be kept in mind with respect to its implication for public health.

## Conclusions

The comparison of patterns of resistance evolution to two classes of drugs against influenza A viruses, Adamantane and Oseltamivir, shows some striking similarities such as multiple emergence of independent mutations and their spread in the human population with negligible role of reassortment, at least within the human host. Although reassortment is known to play a key role among other hosts (Krumbholz et al. 2009) and is an important process in the long-term evolution of H1N1 viruses (Nelson et al. 2008), it is unclear why reassortment has so far played such a negligible role in the emergence and spread of dual resistance in humans. Yet, beyond these similarities, differences exist in the emergence dynamics of these two drugs.

First, the mutations known to cause Oseltamivir resistance are sporadically found in birds but never in any other nonhuman host, whereas Adamantane mutations are found occasionally in birds but are fairly common in swine. In this latter host, whole clades could be found harboring the same mutation, suggesting sustained transmission (Krumbholz et al. 2009). Second, both drug resistances can be caused by multiple mutations, but only in the case of Adamantane could viruses carrying more than one single-drug resistance mutation be found. Third, the first evidence of drug resistance is found almost right after FDA approval for Oseltamivir, but >30 years *before* FDA approval in the case of Adamantane.

Most of these differences are intriguing, even in the case of Adamantane where spontaneous emergence and spread by drift of the S31N mutation have been documented (Hayden and de Jong 2011), even in swine (Krumbholz et al. 2009). Irrespective of the reasons that might have led to these situations, the relative decrease in prevalence of drug resistance mutations both to Adamantane and Oseltamivir hints at a more rational use of these antiviral drugs since the 2009 spread of Adamantane resistance in H1N1. Vigilance should not be lowered however, as dual resistance does exist already in these H1N1 viruses currently circulating in human populations.

## Materials and Methods

### Sequence Retrieval and Alignment

Sensitive, single (Adamantane or Oseltamivir) and dual (Adamantane and Oseltamivir) drug-resistant influenza A/H1N1 human M2 and NA viral genes were first identified from the Influenza Research Database (Squires et al. 2012) using the Phenotypic Characteristic Search for human strains with markers for resistance to Adamantane, Oseltamivir, or both drugs. Complete genomes were then acquired from the IVR (Bao et al. 2008) at NCBI and the Annotation Tool was used to confirm the presence or absence of a given drug resistance mutation. Twenty-nine Adamantane, 20 Oseltamivir, 7 dual-resistant, and 31 sensitive M2 and NA viral gene segments were originally retrieved for the analysis (see supplementary table S1, Supplementary Material online, for accession numbers). As the goal of the study was to date the emergence of drug resistance, more sequences were retrieved to break up long branches along which resistance emerged. These additional sequences were found by performing BlastN searches using the two sequences at the extremities of each branch of interest. The search only included sequences in the years between the long branch, which led us to retrieve an additional 16 human, 5 avian, and 17 swine sequences. The phylogenetic placement of these sequences was assessed by maximum likelihood (ML) with PhyML (Guindon and Gascuel 2003) under the same model of evolution as selected by the procedure described below (see Phylogenetic Analyses). Viral DNA sequences coding for M2 and NA genes were aligned using MUSCLE v3.8.31 (Edgar 2004), and alignment was visualized using Jalview v2.6.1 (Waterhouse et al. 2009).

To place the evolution of drug resistance in a general context, additional sequences were downloaded from the IVR for the segments involved in the two drug resistances and those involved in defining influenza A serotypes: HA, NA, and MP. For these three data sets, all complete nucleotide sequences were downloaded (all hosts, all countries, and all subtypes). Only full-length sequences were retrieved for HA and NA, leading up to data sets comprising 24,168, 20,888, and 19,932 sequences, respectively (HA, NA, and MP, as of July 2013). Two H1N1 data sets were retrieved from the IVR, one containing seasonal (prepandemic 2009) sequences and another one containing pandemic sequences. Sequences isolated from chiropteran hosts (H17) were removed.

### Phylogenetic Analyses

The determination of which model best suited M2 and NA sequences for phylogenetic reconstruction was based on the Akaike information criterion (AIC) as implemented in jModelTest v2.1.3 (Posada 2008). Briefly, likelihood scores were computed for 11 substitutions schemes taking into account base frequencies and rate variation parameters using a BIONJ base tree (Gascuel 1997). AIC calculations were computed for the 88 models returned. For M2 sequences, the GTR+Γ was chosen as a model, whereas for the NA sequences the GTR+I+Γ returned the lowest AIC score and was chosen as a model.

A Bayesian analysis as implemented in BEAST v1.7.5 (Drummond et al. 2012) was used to estimate the phylogeny and date the emergence of drug resistance in M2 and NA. An uncorrelated lognormal relaxed clock prior was placed on rates, whereas a constant size coalescent process was used as a speciation prior. These priors were chosen as the lognormal distribution is more flexible than the exponential distribution, and a coalescent process is appropriate for analyzing population data (e.g., Abdussamad and Aris-Brosou 2011). Sequence collection years were used to calibrate the relaxed clock (Rambaut 2000). For both data sets, four independent Markov chain Monte Carlo (MCMC) chains, each of 100 million steps, were run. A thinning of 5,000 was used to decorrelate samples taken from the MCMC. Tracer (tree.bio.ed.ac.uk/software/tracer) was employed to monitor progress of the MCMC runs, to visually check for convergence and to ensure that removing 25% of the chain as a burn-in was appropriate to sample from the target distributions at stationarity. LogCombiner and TreeAnnotator (both distributed with BEAST) were used to remove the burn-in/combine all four runs for each gene and to generate the maximum a posteriori trees, respectively. Trees were visualized and edited using FigTree (v1.4; tree.bio.ed.ac.uk/software/figtree). Other visualizations and statistical analyses were carried out using R v2.15.2 (R Core Team 2012).

The additional data for HA, NA, and MP were aligned with a local version of TranslatorX (Abascal et al. 2010) customized to use MUSCLE with the fastest heuristics (-maxiters 1 -diags); alignments were sanitized with GBlocks (Castresana 2000) by allowing half of the block positions and extending the maximum number of contiguous nonconserved positions to 24; ML trees were inferred with FastTree (Price et al. 2010) under GTR+Γ. Node support values were estimated following the SH-like aLRT (Anisimova and Gascuel 2006). Trees were plotted with R using the APE library (Paradis et al. 2004); mutations responsible for drug resistance phenotypes (the dominant S31N as well as A30T, G34E, L26F, and V27A for Adamantane; H274Y in N1 as well as E119V, N294S, and R292K in N2 subtypes for Oseltamivir) were extracted with R from sequence alignments and were manually checked against the protein sequences deposited in the IVR.

## Supplementary Material

Supplementary figures S1and S2 and tables S1–S5 are available at *Molecular Biology and Evolution* online (http://www.mbe.oxfordjournals.org/).

Supplementary Data
