# Supplementary material for: Comparative Dynamics and Distribution of Influenza Drug Resistance Acquisition to Protein M2 and Neuraminidase Inhibitors
Source: Mol Biol Evol. 2013 Nov 7;31(2):355–63. doi: 10.1093/molbev/mst204 (PMC3907049; doi:10.1093/molbev/mst204)
Supplement: Supplementary Data [file supp_31_2_355__index.html]

Comparative Dynamics and Distribution of Influenza Drug Resistance Acquisition to Protein M2 and Neuraminidase Inhibitors — Comparative Dynamics and Distribution of Influenza Drug Resistance Acquisition to Protein M2 and Neuraminidase Inhibitors — Supplementary Data 

# Comparative Dynamics and Distribution of Influenza Drug Resistance Acquisition to Protein M2 and Neuraminidase Inhibitors

## Supplementary Data

files

**Files in this Data Supplement:**

- Supplementary Data - pdf file
