# Supplementary material for: Comparative Dynamics and Distribution of Influenza Drug Resistance Acquisition to Protein M2 and Neuraminidase Inhibitors
Source: Mol Biol Evol. 2013 Nov 7;31(2):355–63. doi: 10.1093/molbev/mst204 (PMC3907049; doi:10.1093/molbev/mst204)
Supplement: Supplementary Data [file supp_mst204_130930_vg_sab_si.pdf]

Supplementary tables

Table S1: List of genomes used in this study with their corresponding accession numbers

Resistance phenotype	Strain Name	Origin	PB2	PB1	PA	HA	NP	NA	MP	NS
Adamantane (M2)	A/Alaska/1935	Human	CY019962	CY019961	CY019960	CY019955	CY019958	CY019957	CY019956	CY019959
	A/swine/Belgium/WVL5/1989	Swine	CY037988	CY037989	CY037990	CY037991	CY037992	CY037993	CY037994	CY037995
	A/Boston/DOA22/2011	Human	CY111269	CY111268	CY111267	CY111262	CY111265	CY111264	CY111263	CY111266
	A/District of Columbia/WRAIR0309/2010	Human	CY097834	CY097835	CY097836	CY097837	CY097838	CY097839	CY097840	CY097841
	A/District of Columbia/WRAIR0310/2010	Human	CY097842	CY097843	CY097844	CY097845	CY097846	CY097847	CY097848	CY097849
	A/swine/England/101692/1997	Swine	CY115932	CY115933	CY115934	CY115935	CY115936	CY115937	CY115938	CY115939
	A/swine/England/373/2010	Swine	CY116124	CY116125	CY116126	CY116127	CY116128	CY116129	CY116130	CY116131
	A/swine/England/57610/1999	Swine	CY116219	CY116220	CY116221	CY116222	CY116223	CY116224	CY116225	CY116226
	A/Florida/06/2012	Human	JX905428	JX905427	JX905429	JX905426	JX905430	JX905423	JX905425	JX905424
	A/Hanoi/TX09/2006	Human				AB286012		AB286013	AB286014	
	A/Hawaii/16/2007	Human				EU516059		EU516137	EU516156	
	A/Hawaii/45/2007	Human				EU716531		EU716529	GQ475624	
	A/Henry/1936	Human	CY020452	CY020451	CY020450	CY020445	CY020448	CY020447	CY020446	CY020449
	A/hvPR8/34	Human	EF190971	EF190972	EF190973	EF190974	EF190975	EF190976	EF190977	EF190978
	A/swine/Ille et Vilaine/1455/1999	Swine	CY116377	CY116378	CY116379	CY116380	CY116381	CY116382	CY116383	CY116384
	A/Illinois/NHRC0001/2011	Human	CY092879	CY092878	CY092877	CY092872	CY092875	CY092874	CY092873	CY092876
	A/swine/Italy/670/1987	Swine	CY025260	CY025259	CY025258	CY025253	CY025256	CY025255	CY025254	CY025257
	A/swine/Italy/671/1987	Swine	CY022993	CY022992	CY022991	CY022986	CY022989	CY022988	CY022987	CY022990
	A/Los Angeles/INS423/2010	Human	CY083824	CY083823	CY083822	CY083817	CY083820	CY083819	CY083818	CY083821
	A/Louisiana/03/2009	Human				GQ232067	GQ232066	GQ323570	GQ232065	
	A/Massachusetts/01/2008	Human				EU567000		EU566972	EU716624	
	A/Minnesota/25/2007	Human				EU516094		EU516149	EU716611	
	A/swine/Minsk/1965	Swine							GQ404585	
	A/Nevada/04/2009	Human				GQ338372	GQ457492	GQ338374	GQ338373	GQ457493
	A/New Jersey/1976	Human	CY021964	CY021963	CY021962	CY021957	CY021960	CY021959	CY021958	CY021961
	A/New York/1999	Human	CY062097	CY062096	CY062095	CY062090	CY062093	CY062092	CY062091	CY062094
	A/Rochester/INS599/2011	Human	CY129541		CY129540		CY129538	CY129537	CY129536	
	A/San Diego/WRAIR1648P/2009	Human	CY083301	CY083302	CY083303	CY083304	CY083305	CY083306	CY083307	CY083308

	A/South Carolina/3042/2012	Human				CY130173		CY130174	CY130175	
	A/South Carolina/NHRC0002/2011	Human	CY092903	CY092902	CY092901	CY092896	CY092899	CY092898	CY092897	CY092900
	A/swine/Spain/WVL6/1991	Swine	CY037996	CY037997	CY037998	CY037999	CY038000	CY038001	CY038002	CY038003
	A/swine/Tatarstan/1965	Swine							GQ404586	
	A/Texas/22/2009	Human	GQ338388	GQ160570	GQ457498	GQ160574	GQ160569	GQ160571	GQ160573	GQ160572
	A/Utah/02/2009	Human				GQ323564		GQ894863	GQ894864	
	A/Vermont/03/2009	Human				GQ232007	GQ232004	GQ232005	GQ894814	GQ232006
	A/Washington/10/2008	Human				FJ686964		FJ686963	GQ475723	
Oseltamivir (NA)	A/Alabama/WRAIR1235P/2009	Human	CY100841	CY100842	CY100843	CY100844.2	CY100845	CY100846	CY100847	CY100848
	A/Alaska/02/2009	Human				GQ476017		GQ476018	GQ476019	
	A/Arizona/03/2007	Human				EU516243		EU516196	GQ475614	
	A/Arizona/15/2007	Human				EU566966		EU566968	GQ475636	
	A/California/03/2009	Human				GQ475919		GQ475920	GQ475921	
	A/Florida/18/2008	Human				FJ686987		FJ686986	GQ475769	
	A/Hawaii/01/2008	Human				EU851979		EU851980	GQ475763	
	A/Illinois/15/2007	Human				GQ475641		FJ179363	EU716612	
	A/Illinois/16/2007	Human				GQ475631		FJ179362	EU716565	
	A/Indiana/04/2009	Human				GQ476105		GQ476106	GQ476107	
	A/Maryland/07/2008	Human				GQ475730		GQ475731	GQ475732	
	A/Michigan/05/2008	Human				FJ686997		FJ686996	GQ475803	
	A/Montana/02/2009	Human				GQ476128		GQ476129	GQ476130	
	A/North Carolina/02/2008	Human				EU779626		EU779650	GQ475711	
	A/Ohio/01/2009	Human				GQ475849		GQ475850	GQ475851	
	A/Oklahoma/WRAIR1110P/2009	Human	CY100785	CY100786	CY100787	CY100788.2	CY100789	CY100790	CY100791	CY100792
	A/Oregon/01/2009	Human				GQ476131		GQ476132	GQ476133	
	A/Pennsylvania/13/2007	Human				FJ179360		FJ179361	GQ475619	
	A/Washington/01/2009	Human				GQ338343		GQ338344	GQ475967	
	A/Washington/08/2008	Human				FJ686983		FJ686982	GQ475748	
Dual resistance (M2 and NA)	A/Bethesda/NIH106-D14/2009	Human	HQ263280.2	HQ263279.2	HQ263278.2	HQ263267.2	HQ263276.2	GU571155	HQ263275	HQ263277.2
	A/Bethesda/NIH107-D31/2009	Human	HQ263294.2	HQ263293.2	HQ263292.2	HQ263288.2	HQ263290.2	GU571153	HQ263289	HQ263291.2
	A/Boston/678/2009	Human	CY089322	CY089321	CY089320	CY089315	CY089318	CY089317	CY089316	CY089319
	A/Boston/72/2009	Human	CY080944	CY080943	CY080942	CY080937	CY080940	CY080939	CY080938	CY080941
	A/Kentucky/08/2009	Human				GQ475870		GQ475871	GQ475872	
	A/Texas/38/2009	Human				GQ476031		GQ476032	GQ476033	
	A/West Virginia/02/2009	Human				GQ475882		GQ475883	GQ475884	

Sensitive	A/pintail duck/ALB/219/1977	Avian	CY004481	CY004480	CY004479	CY004474	CY004477	CY004476	CY004475	CY004478
	A/mallard/Maryland/178/2002	Avian	GU050686	GU050685	GU050684	GU050679	GU050682	GU050681	GU050680	GU050683
	A/Arizona/14/1978	Human	CY019970	CY019969	CY019968	CY019963	CY019966	CY019965	CY019964	CY019967
	A/Auckland/586/2000	Human	CY022492	CY022491	CY022490	CY022485	CY022488	CY022487	CY022486	CY022489
	A/Baylor/11735/1982	Human	CY009627	CY009626	CY009625	CY009620	CY009623	CY009622	CY009621	CY009624
	A/Beijing/262/1995	Human	CY033621	CY033620	CY033619	CY033614	CY033617	CY033616	CY033615	CY033618
	A/swine/England/WVL10/1993	Swine	CY037903	CY037904	CY037905	CY037906	CY037907	CY037908	CY037909	CY037910
	A/swine/Belgium/WVL1/1979	Swine		CY037896	CY037897	CY037898	CY037899	CY037900	CY037901	CY037902
	A/Bel/1942	Human	CY009283	CY009282	CY009281	CY009276	CY009279	CY009278	CY009277	CY009280
	A/BH/1935	Human	CY096818	CY096817	CY096816	CY096811	CY096814	CY096813	CY096812	CY096815
	A/Brevig Mission/1/1918	Human	DQ208309	DQ208310	DQ208311	AF116575	AY744935	AF250356	AY130766	AF333238
	A/California/10/1978	Human	CY021724	CY021723	CY021722	CY021717	CY021720	CY021719	CY021718	CY021721
	A/Denver/1957	Human	CY008995	CY008994	CY008993	CY008988	CY008991	CY008990	CY008989	CY008992
	A/swine/Finnistere/2899/1982	Swine				CY116348	CY116349	CY116350	CY116351	CY116352
	A/Fort Monmouth/1-MA/1947	Human	CY087791	CY087790	CY087789	CY087784	CY087787	CY087786	CY087785	CY087788
	A/FortMonmouth/1/47	Human	CY009619	CY009618	CY009617	CY009612	CY009615	CY009614	CY009613	CY009616
	A/swine/France/WVL3/1984	Swine	CY037972	CY037973	CY037974	CY037975	CY037976	CY037977	CY037978	CY037979
	A/swine/France/WVL4/1985	Swine	CY037980	CY037981	CY037982	CY037983	CY037984	CY037985	CY037986	CY037987
	A/Hemsbury/1948	Human	CY077878	CY077879	CY077880	CY077881	CY077882	CY077883	CY077884	CY077885
	A/Hickox/1940	Human	CY013278	CY013277	CY013276	CY013271	CY013274	CY013273	CY013272	CY013275
	A/swine/Hokkaido/2/1981	Swine	AB434389	AB434390	AB434391	AB434392	AB434393	AB434394	AB434395	AB434396
	A/Hong Kong/2637/2004	Human	CY125211	CY125210	CY125209	CY125204	CY125207	CY125206	CY125205	CY125208
	A/Iowa/1943	Human	CY020468	CY020467	CY020466	CY020461	CY020464	CY020463	CY020462	CY020465
	A/swine/Iowa/2/1986	Swine	CY096890	CY096889	CY096888	CY096883	CY096886	CY096885	CY096884	CY096887
	A/Johannesburg/159/1997	Human	CY125075	CY125074	CY125073	CY125068	CY125071	CY125070	CY125069	CY125072
	A/Lackland/3/1978	Human	CY020172	CY020171	CY020170	CY020165	CY020168	CY020167	CY020166	CY020169
	A/Malaysia/14075/1997	Human	CY119105	CY119104	CY119103	CY119098	CY119101	CY119100	CY119099	CY119102
	A/Memphis/1/1984	Human	CY021732	CY021731	CY021730	CY021725	CY021728	CY021727	CY021726	CY021729
	A/turkey/MO/21939/1987	Avian	EU743150	EU743149	EU743148	EU743143	EU743146	EU743145	EU743144	EU743147
	A/Netherlands/001S1/1948	Human	CY077753	CY077754	CY077755	CY077756	CY077757	CY077758	CY077759	CY077760
	A/Netherlands/002K1/1949	Human	CY077761	CY077762		CY077763	CY077764	CY077765	CY077766	CY077767
	A/New York/223/2003	Human	CY002695	CY002694	CY002694	CY002688	CY002691	CY002690	CY002689	CY002692
	A/New York/2924-1/1986	Human	CY021740	CY021739	CY021738	CY021733	CY021736	CY021735	CY021734	CY021737
	A/New York/443/2001	Human	CY003479	CY003478	CY003477	CY003472	CY003475	CY003474	CY003473	CY003476
	A/Ostrava/801/1998	Human	CY125099	CY125098	CY125097	CY125092	CY125095	CY125094	CY125093	CY125096
	A/Phila/1935	Human	CY020476	CY020475	CY020474	CY020469	CY020472	CY020471	CY020470	CY020473

A/South Carolina/1/1918	Human				AF117241	AH006859	U94893	U94897	
A/swine/Tennessee/79/1977	Swine	CY022300	CY022299	CY022298	CY022293	CY022296	CY022295	CY022294	CY022297
A/Texas/2922-3/1986	Human	CY020572	CY020571	CY020570	CY020565	CY020568	CY020567	CY020566	CY020569
A/United Kingdom/1-MA/1933	Human	CY090852	CY090851	CY090850	CY090845	CY090848	CY090847	CY090846	CY090849
A/Wisconsin/3523/1988	Human		M25934			M63755	U47816	M63521	
A/duck/Victoria/23/1981	Avian	CY077684	CY077683	CY077682	CY077677	CY077680	CY077679	CY077678	CY077681
A/Wellington/47/1992	Human	CY125043	CY125042	CY125041	CY125036	CY125039	CY125038	CY125037	CY125040

Table S2. Sequences with drug-resistant mutations other than H274Y (Oseltamivir: NA) across all subtypes and all hosts.

Mutation	Sequence list
E119V	A/Tokyo/2101/2004_H3N2, A/Fukui/45/2004_H3N2, A/Mississippi/05/2011_H3N2, A/Mississippi/05/2011_H3N2, A/Texas/12/2007_H3N2, A/Texas/12/2007_H3N2, A/Memphis/15d/2008_H3N2, A/Memphis/4/2002_H3N2, A/Memphis/5/2005_H3N2, A/chicken/Taiwan/SP1/00_H6N1
N294S	A/duck/EasternChina/40/2003_H11N2, A/goose/Guangdong/k0103/2010_H5N5 [N291S], A/Taiwan/30017/2002_H1N1 [N295S], A/turkey/Egypt/7/2007_H5N1, A/Duck/HongKong/380_5/2001_H5N1, A/HongKong/378_1/2001_H5N1, A/duck/Zhejiang/bj/2002_H5N1, A/chicken/India/82616/2008_H5N1, A/chicken/India/85459/2008_H5N1, A/chicken/India/82544/2008_H5N1, A/Egypt/14725_NAMRU3/2006_H5N1, A/Egypt/14724_NAMRU3/2006_H5N1, A/Egypt/N11981/2009_H5N1, A/Managua/171.01/2009_H1N1* [N182S], A/Managua/5590.02/2009_H1N1* [N182S]
R292K	A/Bethesda/956/2006_H3N2, A/Okayama/23/2004_H3N2, A/England/442/2003_H3N2, A/Taiwan/S02076/2013_H7N9 [R289K], A/quail/HongKong/FY119/2001_H6N1 [R274K]

Notes—Homologous positions for drug-resistant mutations are indicated in square brackets where different from the reference positions. *: H1N1 pandemic sequences.

Table S3. Sequences with multiple drug-resistant mutations (Adamantane: MP).

Mutations	Sequence list
S31N & L26F	A/swine/Saitama/21/2004_H1N2, A/swine/Italy/1081/00_H1N2, A/swine/Italy/18/2000_H1N2, A/swine/England/60797/2000_H1N2, A/swine/England/661263/1997_H1N2, A/swine/England/68327/1998_H1N1, A/swine/Cotesd'Armor/790/97_H1N2, A/swine/Cotesd'Armor/790/1997_H1N2, A/swine/Italy/1654-1/99_H1N2, A/swine/Cotesd'Armor/1482/1999_H1N1, A/swine/Italy/1521/98_H1N2, A/swine/Italy/62/1998_H1N2, A/swine/England/WVL14/1996_H1N1, A/Connecticut/02/2010_H1N1*, A/Bangkok/INS587/2010_H1N1*, A/KhonKaen/INS588/2010_H1N1*, A/swine/Illinois/A01241208/2012_H3N2, A/swine/Illinois/A01240575/2011_H3N2, A/swine/Illinois/A01241212/2012_H3N2, A/swine/Illinois/A01241065/2012_H3N2, A/Scotland/Glasgow_444809/2009_H1N1*, A/swine/PinardelRio/3/2010_H1N1*, A/Mum/NIV9945/2009_H1N1*
S31N & V27A	A/swine/Aomori/1/2005_H1N2, A/PuertoRico/8-LVD3/1934_H1N1, A/PAL/unknown_H1N2, A/PuertoRico/8/34/MountSinai_H1N1, A/Shaanxi-Beilin/1106/2007_H1N1, A/Australia/18/2009_H3N2, A/Australia/34/2009_H3N2, A/California/VRDL204/2009_H3N2, A/Australia/22/2009_H3N2, A/NewYork/3542/2009_H3N2, A/Delaware/04/2010_H3N2, A/Colorado/UR06-0023/2007_H3N2, A/Habana/322/2013_H3N2, A/Beijing-Xicheng/148/2007_H3N2, A/swine/OMS/2112/1995_H1N1, A/swine/England/483/2006_H1N2, A/swine/Schwerin/103/89_H1N1, A/chicken/WestJava/Smi-Biot/2008_H5N1, A/chicken/WestJava/Smi-M6/2008_H5N1, A/chicken/WestJava/Smi-M1/2008_H5N1, A/Indonesia/CDC357/2006_H5N1, A/duck/VietNam/DT-9/2007_H5N1, A/duck/CaoBang/43/2007_H5N1, A/duck/Vietnam/NCVD-43/2007_H5N1, A/duck/Vietnam/37/2007_H5N1, A/chicken/NamDinh/07-32/2007_H5N1, A/duck/Vietnam/NCVD-19/2007_H5N1, A/chicken/Malaysia/5858/2004_H5N1, A/chicken/Bangladesh/12VIR-7140-5/2012_H5N1, A/chicken/Taiwan/0329/01_H6N1, A/chicken/WestJava/Smi-

	Hj18/2007_H5N1, A/chicken/WestJava/Smi-Sud1/2007_H5N1, A/chicken/Cambodia/67F8/2008_H5N1, A/Indonesia/CDC1046/2007_H5N1, A/chicken/Cambodia/67F4/2008_H5N1
S31N & A30T	A/Sydney/DD3-51/2010_H1N1*
S31N & G34E	A/swine/Alberta/OTH-33-23/2009_H1N1*

Notes—*: H1N1 pandemic sequences.

Table S4. Sequences with Adamantane drug-resistant mutations collected before FDA approval of Adamantane (1966).

Mutation	Sequence list
S31N	A/swine/Minsk/1965_H1N1, A/PuertoRico/8/1934_H1N1, A/Alaska/1935_H1N1, A/PuertoRico/8-LVD3/1934_H1N1, A/Henry/1936_H1N1, A/PuertoRico/8/1934_H1N1, A/PuertoRico/8/1934_H1N1, A/PuertoRico/8-1/1934_H1N1, A/PuertoRico/8-SV14/1934_H1N1, A/PuertoRico/8/1934_H1N1, A/PuertoRico/8-WG/1934_H1N1, A/PuertoRico/8/1934_H1N1, A/PuertoRico/8/1934_H1N1, A/AA/Huston/1945_H1N1, A/Weiss/1943_H1N1, A/Melbourne/1/1946_H1N1, A/Melbourne/1935_H1N1, A/WSN/1933_H1N1, A/chicken/Brescia/1902_H7N7, A/WSN/1933_H1N12, A/Wilson-Smith/1933_H1N1, A/WSN/1933_H1N1, A/NWS/1934_H1N1, A/NWS/1933_H1N1, A/WS/7/1933_H1N1, A/Wilson-Smith/1933_H1N1, A/tern/SouthAfrica/1959_H5N3, A/tern/SouthAfrica/1961_H5N3, A/WSN/1933_H1N14, A/WSN/1933_H1N1
V27A	A/PuertoRico/8/1934_H1N1, A/PuertoRico/8-LVD3/1934_H1N1
L26F	None
A30T	None
G34E	A/WSN/1933_H1N1

Table S5. Sequences with drug-resistant mutation H274Y (Oseltamivir: NA) across all subtypes and all hosts.

Sequence list
A/Illinois/03/2012_H1N1,
A/Texas/26/2012_H1N1,
A/Texas/49/2012_H1N1,
A/Texas/48/2012_H1N1,
A/Texas/33/2012_H1N1,
A/California/21/2012_H1N1,
A/Texas/30/2012_H1N1,
A/Mexico/InDRE1474/2012_H1N1,
A/Mexico/InDRE5726/2012_H1N1,
A/Mexico/InDRE6023/2012_H1N1,
A/Taiwan/652/2011_H1N1,
A/Washington/24/2012_H1N1,
A/Texas/35/2012_H1N1,
A/Texas/34/2012_H1N1,
A/Texas/31/2012_H1N1,
A/Illinois/01/2012_H1N1,
A/NewYork/34/2012_H1N1,
A/Sydney/JP2Y/2011_H1N1,
A/Sydney/0794/2011_H1N1,
A/Sydney/CB1/2011_H1N1,
A/Taiwan/549/2011_H1N1,
A/Kyoto/10K104_2/2011_H1N1,
A/Kyoto/10K124/2011_H1N1,
A/Hyogo/10K291/2011_H1N1,
A/Thailand/CU_H2358/2010_H1N1,
A/Sydney/CH2/2011_H1N1,
A/Taiwan/1758/2011_H1N1,
A/Taiwan/2116/2011_H1N1,
A/Maryland/06/2011_H1N1,
A/Colorado/11/2010_H1N1,
A/Delaware/08/2011_H1N1,
A/Parana/409/2012_H1N1,
A/Washington/01/2010_H1N1,
A/Washington/29/2009_H1N1,
A/Maine/04/2011_H1N1,

A/Delaware/07/2011_H1N1,
A/St_Petersburg/CRIE_GoVM/2011_H1N1,
A/Thailand/CU_H2283/2010_H1N1,
A/Thailand/CU_H2389/2010_H1N1,
A/Singapore/GP4344/2010_H1N1,
A/Sydney/DD3_48/2010_H1N1,
A/Taiwan/1829/2011_H1N1,
A/Taiwan/4709/2011_H1N1,
A/Taiwan/4711/2011_H1N1,
A/Singapore/TT450/2010_H1N1,
A/Singapore/TT454/2010_H1N1,
A/Cologne/INS537/2011_H1N1,
A/Munich/INS541/2011_H1N1,
A/Sheffield/INS545/2011_H1N1,
A/Delaware/06/2011_H1N1,
A/Maryland/07/2011_H1N1,
A/Indiana/04/2011_H1N1,
A/Maryland/08/2011_H1N1,
A/RioGrandeDoSul/687/2012_H1N1,
A/SantaCatarina/229/2012_H1N1,

A/Maryland/04/2011_H1N1,

A/Singapore/EN103/2010_H1N1,

A/Lebanon/09L_22/2009_H1N1,
A/England/94640080/2009_H1N1,
A/Seoul/1870/2009_H1N1,
A/Taiwan/6663/2009_H1N1,
A/Ontario/10296/2009_H1N1,

A/Ontario/25913/2009_H1N1,

A/Washington/28/2009_H1N1,
A/HongKong/2369/2009_H1N1,
A/Osaka/180/2009_H1N1,
A/Denmark/528/2009_H1N1,
A/Hunan/SWL3/2009_H1N1,
A/Tokushima/2/2009_H1N1,
A/Taiwan/7717/2009_H1N1,
A/Utah/34/2009_H1N1,
A/Texas/48/2009_H1N1,
A/Russia/61/2009_H1N1,
A/Bethesda/NIH106_D14/2009_H1N1,
A/Singapore/GN285/2009_H1N1,
A/Taiwan/7855/2009_H1N1,
A/Taiwan/7336/2009_H1N1,
A/Taiwan/2343/2009_H1N1,
A/Seoul/1785/2009_H1N1,

A/Haishu/SWL110/2010_H1N1,
A/Singapore/GP562/2010_H1N1,
A/Mexicocity/CIA10/2009_H1N1,
A/Netherlands/2445b/2009_H1N1,
A/Netherlands/2290/2009_H1N1,
A/Brussels/INS244/2009_H1N1,
A/Lisboa/171/2009_H1N1,
A/Athens/INS417/2010_H1N1,
A/Hokkaido/256/2009_H1N1,
A/Argentina/HNRG23/2009_H1N1,
A/Lyon/48_425/2009_H1N1,
A/Lyon/48_49/2009_H1N1,
A/HongKong/23369/2009_H1N1,
A/Boston/678/2009_H1N1,
A/Kyoto/09K266_2/2009_H1N1,
A/Kyoto/09K328_2/2009_H1N1,
A/Taiwan/1240/2011_H1N1,
A/Yamaguchi/248/2009_H1N1,
A/England/93520038/2009_H1N1,
A/England/92960004/2009_H1N1,
A/VietNam/817/2009_H1N1,
A/VietNam/835/2009_H1N1,
A/VietNam/12032005/2009_H1N1,
A/NorthCarolina/59/2009_H1N1,
A/Tennessee/17/2009_H1N1,
A/Georgia/23/2009_H1N1,
A/Illinois/10/2009_H1N1,
A/California/38/2009_H1N1,

A/Mexico/InDRE797/2010_H1N1,

A/Nagasaki/HA_58/2009_H1N1,

A/Yamagata/51/2009_H1N1,

A/KOL/2449/2009_H1N1,
A/Tehran/310/2009_H1N1,
A/England/594/2006_H1N1,
A/Delaware/01/2009_H1N1,
A/Managua/954_02/2008_H1N1,
A/Managua/107_01/2008_H1N1,
A/England/684/2007_H1N1,
A/Florida/02/2008_H1N1,

A/Hawaii/21/2007_H1N1,

A/Hawaii/28/2007_H1N1,
A/Scotland/5/2007_H1N1,
A/England/654/2007_H1N1,

A/Victoria/07159220/2007_H1N1,
A/Managua/3153_01/2008_H1N1,
A/Hawaii/28/2007_H1N1,
A/Montana/02/2009_H1N1,
A/Hyogo/07K030/2007_H1N1,
A/Nagasaki/07N020/2008_H1N1,
A/Yamagata/66/2008_H1N1,
A/Taiwan/3293/2008_H1N1,
A/Nevada/WRAIR1257P/2009_H1N1,
A/Nevada/WRAIR1117P/2009_H1N1,
A/Tehran/345/2009_H1N1,
A/HongKong/62768/2008_H1N1,
A/Kericho/7529/2008_H1N1,
A/Peru/WRAIR1310P/2007_H1N1,
A/Maracay/WRAIR1729P/2008_H1N1,
A/Pennsylvania/04/2009_H1N1,
A/Hue/H445/2008_H1N1,
A/Taiwan/0045/2006_H1N1,
A/KOL/2465/2009_H1N1,
A/Novosibirsk/3/2009_H1N1,
A/Thailand/CU_B267/2009_H1N1,
A/Virginia/06/2009_H1N1,
A/NewJersey/32/2008_H1N1,
A/Philippines/1159/2008_H1N1,
A/Arizona/13/2008_H1N1,
A/California/VRDL371/2009_H1N1,
A/Kiev/313/2008_H1N1,
A/Boston/17/2009_H1N1,
A/Boston/95/2009_H1N1,
A/Pucallpa/WRAIR1704P/2009_H1N1,
A/Peru/WRAIR1298P/2007_H1N1,
A/Piura/WRAIR1699P/2008_H1N1,
A/Piura/WRAIR1700P/2009_H1N1,
A/Pucallpa/WRAIR1709P/2009_H1N1,
A/Trujillo/WRAIR1702P/2008_H1N1,
A/Boston/46/2009_H1N1,
A/Peru/WRAIR1295P/2007_H1N1,
A/Peru/WRAIR1299P/2007_H1N1,
A/Piura/WRAIR1708P/2009_H1N1,

A/Yamagata/45/2009_H1N1,

A/Niigata/08F031/2009_H1N1,

A/Kawasaki/UTK_4/2009_H1N1,
A/Maryland/01/2009_H1N1,
A/Maryland/04/2009_H1N1,
A/RhodeIsland/01/2009_H1N1,
A/NewYork/01/2009_H1N1,

A/Luxembourg/116/2008_H1N1,
A/Wisconsin/12/2008_H1N1,
A/Massachusetts/09/2008_H1N1,
A/Florida/28/2008_H1N1,
A/NewYork/65/2009_H1N1,
A/Hawaii/17/2009_H1N1,
A/Pennsylvania/12/2009_H1N1,
A/NewMexico/01/2009_H1N1,

A/California/VRDL213/2009_H1N1,

A/California/VRDL236/2009_H1N1,
A/California/VRDL245/2009_H1N1,
A/Boston/49/2009_H1N1,
A/Boston/88/2009_H1N1,
A/Boston/87/2009_H1N1,
A/Illinois/05/2009_H1N1,
A/Maryland/04/2008_H1N1,
A/Johannesburg/21/2008_H1N1,
A/Johannesburg/28/2008_H1N1,
A/Ohio/01/2009_H1N1,
A/Ohio/06/2009_H1N1,
A/Boston/72/2009_H1N1,
A/Oklahoma/WRAIR1110P/2009_H1N1,
A/Peru/WRAIR1300P/2007_H1N1,
A/Texas/WRAIR1115P/2009_H1N1,
A/Washington/WRAIR1237P/2009_H1N1,
A/California/VRDL175/2009_H1N1,
A/California/VRDL270/2009_H1N1,
A/Thailand/778/2008_H1N1,
A/Thailand/669/2008_H1N1,
A/Nebraska/07/2008_H1N1,
A/Illinois/16/2008_H1N1,

A/Illinois/17/2008_H1N1,

A/Illinois/15/2008_H1N1,
A/Delaware/01/2008_H1N1,
A/Illinois/12/2009_H1N1,
A/Texas/57/2009_H1N1,
A/RhodeIsland/17/2009_H1N1,
A/Malaysia/2132379/2008_H1N1,
A/Malaysia/2089302/2009_H1N1,
A/DaNang/DN431/2008_H1N1,
A/DaNang/DN467/2008_H1N1,
A/Hue/H433/2008_H1N1,
A/SantaCruz/WRAIR1706P/2008_H1N1,
A/Trujillo/WRAIR1701P/2008_H1N1,
A/Sullana/WRAIR1707P/2009_H1N1,

A/Iraq/WRAIR1683P/2009_H1N1,
A/Alabama/WRAIR1235P/2009_H1N1,
A/Texas/WRAIR1118P/2009_H1N1,
A/Cusco/WRAIR1730P/2008_H1N1,
A/Texas/WRAIR1053P/2009_H1N1,
A/Boston/3/2009_H1N1,
A/Boston/44/2009_H1N1,
A/Boston/85/2009_H1N1,
A/Boston/67/2009_H1N1,
A/Boston/52/2009_H1N1,
A/Tottori/52/2008_H1N1,
A/Taiwan/10103/2008_H1N1,
A/Taiwan/11526/2008_H1N1,
A/NorthCarolina/04/2009_H1N1,
A/California/VRDL249/2009_H1N1,
A/California/VRDL232/2009_H1N1,
A/California/VRDL202/2009_H1N1,
A/California/VRDL200/2009_H1N1,
A/California/VRDL180/2009_H1N1,
A/California/VRDL251/2009_H1N1,
A/Myanmar/M533/2009_H1N1,
A/Myanmar/M428/2009_H1N1,
A/California/VRDL167/2009_H1N1,
A/California/VRDL221/2009_H1N1,
A/California/VRDL237/2009_H1N1,
A/Kisii/7541/2008_H1N1,
A/Kisii/7547/2008_H1N1,
A/Bogota/WRAIR0088N/2009_H1N1,
A/Yamagata/77/2009_H1N1,
A/Yamagata/16/2009_H1N1,
A/Yamagata/26/2009_H1N1,

A/Yamagata/125/2008_H1N1,

A/Novosibirsk/716/2009_H1N1,

A/Gunma/08G006/2009_H1N1,

A/Novosibirsk/4/2009_H1N1,

A/Novosibirsk/151/2009_H1N1,

A/Indiana/03/2009_H1N1,
A/Oregon/01/2009_H1N1,
A/Hawaii/01/2009_H1N1,
A/Pennsylvania/01/2009_H1N1,
A/NewYork/3442/2009_H1N1,
A/Nebraska/05/2009_H1N1,
A/Arkansas/02/2009_H1N1,

A/Illinois/06/2009_H1N1,
A/Arkansas/01/2009_H1N1,
A/Texas/38/2009_H1N1,
A/Pennsylvania/11/2009_H1N1,
A/NorthDakota/01/2009_H1N1,
A/NewYork/63/2009_H1N1,
A/Maryland/03/2009_H1N1,
A/Hawaii/04/2009_H1N1,
A/Kentucky/01/2009_H1N1,
A/SouthDakota/02/2009_H1N1,
A/WestVirginia/02/2009_H1N1,
A/Tennessee/01/2009_H1N1,
A/Hawaii/04/2009_H1N1,
A/Texas/36/2008_H1N1,
A/NewJersey/30/2008_H1N1,
A/Washington/03/2009_H1N1,
A/Hawaii/58/2008_H1N1,
A/Pennsylvania/12/2008_H1N1,
A/Pennsylvania/14/2008_H1N1,
A/NewJersey/01/2008_H1N1,
A/Saskatchewan/5131/2009_H1N1,
A/Saskatchewan/5350/2009_H1N1,
A/Saskatchewan/5351/2009_H1N1,
A/Thailand/1035/2008_H1N1,
A/Washington/01/2009_H1N1,
A/Vermont/03/2008_H1N1,
A/Managua/4924_01/2008_H1N1,
A/Managua/5007_01/2008_H1N1,
A/Johannesburg/37/2008_H1N1,
A/Johannesburg/279/2008_H1N1,
A/Texas/27/2008_H1N1,
A/NewMexico/02/2009_H1N1,
A/Managua/496_01/2008_H1N1,
A/Managua/1985_01/2008_H1N1,
A/California/VRDL377/2009_H1N1,
A/California/VRDL378/2009_H1N1,
A/Kuwait/WRAIR1119P/2009_H1N1,
A/Pennsylvania/08/2008_H1N1,
A/Utah/12/2009_H1N1,
A/Illinois/WRAIR1233P/2009_H1N1,
A/Texas/17/2008_H1N1,
A/Pennsylvania/09/2008_H1N1,
A/Texas/18/2008_H1N1,
A/Texas/15/2008_H1N1,
A/Texas/16/2008_H1N1,
A/Texas/23/2008_H1N1,
A/Hawaii/20/2008_H1N1,
A/Texas/29/2008_H1N1,

A/Hawaii/20/2008_H1N1,
A/Hawaii/42/2008_H1N1,
A/Hawaii/55/2008_H1N1,
A/Hawaii/38/2008_H1N1,

A/Colorado/08/2008_H1N1,

A/Hawaii/59/2008_H1N1,
A/Texas/35/2008_H1N1,
A/Texas/32/2008_H1N1,
A/Hawaii/20/2008_H1N1,
A/NewYork/02/2009_H1N1,
A/Nebraska/06/2009_H1N1,
A/Indiana/05/2009_H1N1,
A/Kansas/01/2009_H1N1,
A/Hawaii/03/2009_H1N1,
A/Texas/01/2009_H1N1,
A/Ohio/04/2009_H1N1,
A/Wisconsin/06/2009_H1N1,
A/Indiana/11/2009_H1N1,

A/Arizona/10/2009_H1N1,

A/Nebraska/01/2009_H1N1,
A/Hawaii/03/2009_H1N1,
A/Hawaii/13/2009_H1N1,
A/Florida/02/2009_H1N1,

A/Wisconsin/13/2009_H1N1,

A/Alaska/01/2009_H1N1,

A/Managua/1409_01/2008_H1N1,

A/California/VRDL140/2009_H1N1,
A/Texas/WRAIR1052P/2009_H1N1,
A/SouthDakota/WRAIR1113P/2009_H1N1,
A/Alaska/WRAIR1114P/2009_H1N1,
A/Louisiana/WRAIR1170P/2009_H1N1,
A/California/WRAIR1243P/2009_H1N1,
A/Arkansas/WRAIR1249P/2009_H1N1,
A/Texas/WRAIR1254P/2009_H1N1,
A/California/VRDL149/2009_H1N1,
A/California/VRDL154/2009_H1N1,
A/California/VRDL163/2009_H1N1,
A/California/VRDL218/2009_H1N1,
A/California/VRDL235/2009_H1N1,
A/California/VRDL238/2009_H1N1,
A/California/VRDL252/2009_H1N1,
A/California/VRDL258/2009_H1N1,

A/California/VRDL290/2009_H1N1,
A/California/VRDL292/2009_H1N1,
A/California/VRDL295/2009_H1N1,
A/California/VRDL301/2009_H1N1,
A/California/VRDL379/2009_H1N1,
A/Boston/76/2009_H1N1,
A/Boston/19/2009_H1N1,
A/NewYork/3150/2009_H1N1,
A/Pennsylvania/11/2009_H1N1,
A/Hawaii/13/2009_H1N1,
A/Austria/406109/2008_H1N1,
A/Austria/404811/2008_H1N1,
A/Kentucky/08/2009_H1N1,
A/Niigata/08F188/2009_H1N1,
A/Norway/1736/2007_H1N1,
A/Johannesburg/45/2008_H1N1,
A/Johannesburg/14/2008_H1N1,
A/Johannesburg/35/2008_H1N1,
A/Victoria/501/2008_H1N1,
A/Perth/46/2008_H1N1,
A/Sydney/7/2008_H1N1,
A/NewCaledonia/6/2008_H1N1,
A/Washington/05/2008_H1N1,
A/Indiana/01/2008_H1N1,
A/Maryland/04/2007_H1N1,
A/Arizona/03/2007_H1N1,

A/Gyeonggi/161/2009_H1N1,

A/NewJersey/15/2007_H1N1,
A/Illinois/10/2007_H1N1,
A/NewJersey/16/2007_H1N1,
A/Pennsylvania/02/2008_H1N1,
A/Arizona/14/2007_H1N1,
A/NewJersey/06/2008_H1N1,
A/England/557/2007_H1N1,
A/Washington/01/2008_H1N1,
A/Memphis/03/2008_H1N1,
A/NorthCarolina/02/2008_H1N1,
A/Hawaii/01/2008_H1N1,
A/Johannesburg/36/2008_H1N1,
A/Pennsylvania/13/2007_H1N1,
A/Illinois/16/2007_H1N1,
A/Illinois/15/2007_H1N1,
A/Athens/41/2008_H1N1,
A/Lyon/1337/2007_H1N1,
A/Belgium/G257/2008_H1N1,
A/Slovenia/123/2008_H1N1,

A/Canarias/RR_2915/2007_H1N1,

A/Denmark/27/2007_H1N1,

A/England/26/2008_H1N1,
A/Hawaii/19/2008_H1N1,
A/Wisconsin/17/2008_H1N1,
A/Maryland/05/2008_H1N1,
A/Massachusetts/08/2008_H1N1,
A/Tennessee/11/2008_H1N1,
A/Washington/08/2008_H1N1,
A/Florida/24/2008_H1N1,
A/NorthDakota/01/2008_H1N1,
A/Minnesota/27/2008_H1N1,
A/Florida/21/2008_H1N1,
A/Massachusetts/08/2008_H1N1,
A/Michigan/06/2008_H1N1,
A/NewJersey/23/2008_H1N1,
A/Wisconsin/16/2008_H1N1,
A/SouthAustralia/1/2008_H1N1,
A/Perth/33/2008_H1N1,
A/Yangon/M195/2008_H1N1,
A/HongKong/17/2008_H1N1,
A/HongKong/349/2008_H1N1,
A/Uruguay/03/2008_H1N1,
A/Iowa/01/2009_H1N1,
A/HongKong/17/2008_H1N1,
A/HongKong/349/2008_H1N1,
A/Uruguay/03/2008_H1N1,
A/Hawaii/19/2008_H1N1,
A/Iowa/01/2009_H1N1,
A/NorthCarolina/07/2008_H1N1,

A/Colorado/17/2008_H1N1,

A/Michigan/10/2008_H1N1,
A/Minnesota/34/2008_H1N1,
A/Wyoming/10/2008_H1N1,
A/Wisconsin/20/2008_H1N1,
A/Minnesota/33/2008_H1N1,
A/NewJersey/20/2008_H1N1,
A/Pennsylvania/14/2008_H1N1,
A/Wisconsin/04/2009_H1N1,
A/SouthDakota/01/2009_H1N1,
A/Wisconsin/02/2009_H1N1,
A/NewYork/62/2009_H1N1,
A/Wisconsin/01/2009_H1N1,
A/California/03/2009_H1N1,
A/Virginia/03/2009_H1N1,

A/Indiana/07/2009_H1N1,
A/Montana/03/2009_H1N1,
A/Kentucky/03/2009_H1N1,
A/California/02/2009_H1N1,
A/Montana/01/2009_H1N1,
A/Oregon/02/2009_H1N1,
A/Massachusetts/05/2009_H1N1,
A/Minnesota/01/2009_H1N1,
A/NorthDakota/03/2009_H1N1,

A/Michigan/07/2009_H1N1,

A/Florida/03/2009_H1N1,

A/Tennessee/02/2009_H1N1,
A/Minnesota/01/2009_H1N1,
A/NewYork/56/2009_H1N1,
A/Ohio/03/2009_H1N1,
A/Florida/07/2009_H1N1,

A/Vermont/01/2009_H1N1,

A/Minnesota/04/2009_H1N1,
A/NewYork/64/2009_H1N1,
A/Shanghai/LWS1/2009_H1N1,
A/Wisconsin/14/2009_H1N1,
A/Thailand/CU_B42/2009_H1N1,
A/Thailand/CU_H17/2009_H1N1,
A/Thailand/829/2008_H1N1,
A/Tehran/359/2009_H1N1,
A/NewYork/1692/2009_H1N1,
A/NewYork/3095/2009_H1N1,
A/NewYork/3315/2009_H1N1,
A/Taiwan/9042/2008_H1N1,
A/Thailand/CU_B589/2009_H1N1,
A/Thailand/CU_B685/2009_H1N1,
A/Thailand/CU_H223/2009_H1N1,
A/Thailand/CU_B97/2009_H1N1,
A/Thailand/CU_H565/2009_H1N1,
A/Novosibirsk/2/2009_H1N1,

A/Novosibirsk/7/2009_H1N1,

A/Novosibirsk/8/2009_H1N1,

A/Nagasaki/07N035/2008_H1N1,

A/Niigata/08F093/2009_H1N1,

A/Hokkaido/08H024/2009_H1N1,

A/Kyoto/08K056/2009_H1N1,
A/Nagasaki/08N006/2009_H1N1,

A/Tottori/08T010/2008_H1N1,

A/Yokohama/78/2008_H1N1,

A/Yamagata/126/2008_H1N1,

A/Yamagata/128/2008_H1N1,

A/Yamagata/137/2008_H1N1,

A/Yamagata/36/2009_H1N1,

A/Yamagata/53/2009_H1N1,

A/Yamagata/55/2009_H1N1,

A/Yamagata/61/2009_H1N1,

A/Yamagata/80/2009_H1N1,

A/NewJersey/15/2007_H1N1,

A/California/VRDL134/2009_H1N1,

A/California/VRDL141/2009_H1N1,

A/California/VRDL146/2009_H1N1,

A/California/VRDL135/2009_H1N1,

A/Singapore/GP101/2009_H1N1,
A/SouthCarolina/WRAIR1112P/2009_H1N1,
A/SouthCarolina/WRAIR1116P/2009_H1N1,
A/Washington/WRAIR1126P/2009_H1N1,
A/Japan/WRAIR1230P/2009_H1N1,
A/Nebraska/WRAIR1247P/2009_H1N1,
A/California/VRDL150/2009_H1N1,
A/California/VRDL151/2009_H1N1,
A/Mbagathi/7596/2008_H1N1,
A/California/VRDL152/2009_H1N1,
A/California/VRDL153/2009_H1N1,
A/California/VRDL155/2009_H1N1,
A/California/VRDL229/2009_H1N1,
A/Myanmar/M061/2009_H1N1,
A/Myanmar/M218/2009_H1N1,
A/Myanmar/M256/2009_H1N1,
A/Myanmar/M278/2009_H1N1,
A/Myanmar/M286/2009_H1N1,
A/Myanmar/M374/2009_H1N1,

A/Myanmar/M470/2009_H1N1,
A/California/VRDL186/2009_H1N1,
A/California/VRDL189/2009_H1N1,
A/California/VRDL192/2009_H1N1,
A/California/VRDL193/2009_H1N1,
A/California/VRDL194/2009_H1N1,
A/California/VRDL206/2009_H1N1,
A/California/VRDL209/2009_H1N1,
A/California/VRDL217/2009_H1N1,
A/California/VRDL220/2009_H1N1,
A/California/VRDL222/2009_H1N1,
A/California/VRDL223/2009_H1N1,
A/California/VRDL224/2009_H1N1,
A/California/VRDL226/2009_H1N1,
A/California/VRDL254/2009_H1N1,
A/California/VRDL256/2009_H1N1,
A/California/VRDL264/2009_H1N1,
A/California/VRDL267/2009_H1N1,
A/California/VRDL273/2009_H1N1,
A/California/VRDL275/2009_H1N1,
A/California/VRDL276/2009_H1N1,
A/California/VRDL280/2009_H1N1,
A/California/VRDL281/2009_H1N1,
A/California/VRDL283/2009_H1N1,
A/California/VRDL284/2009_H1N1,
A/California/VRDL289/2009_H1N1,
A/California/VRDL300/2009_H1N1,
A/California/VRDL348/2009_H1N1,
A/California/VRDL358/2009_H1N1,
A/California/VRDL365/2009_H1N1,
A/California/VRDL366/2009_H1N1,
A/California/VRDL369/2009_H1N1,
A/California/VRDL380/2009_H1N1,
A/California/VRDL382/2009_H1N1,
A/Taiwan/2885/2008_H1N1,
A/Taiwan/5857/2008_H1N1,
A/Taiwan/8949/2008_H1N1,
A/Taiwan/1026/2009_H1N1,
A/Tianjin/15/2009_H1N1,
A/Boston/1/2009_H1N1,
A/Boston/11/2009_H1N1,
A/Boston/13/2009_H1N1,
A/Boston/14/2009_H1N1,
A/Boston/18/2009_H1N1,
A/Boston/20/2009_H1N1,
A/Boston/24/2009_H1N1,
A/Boston/30/2009_H1N1,
A/Boston/36/2009_H1N1,

A/Boston/39/2009_H1N1,
A/Boston/41/2009_H1N1,
A/Boston/42/2009_H1N1,
A/Boston/43/2009_H1N1,
A/Boston/45/2009_H1N1,
A/Boston/48/2009_H1N1,
A/Boston/51/2009_H1N1,
A/Boston/58/2009_H1N1,
A/Boston/62/2009_H1N1,
A/Boston/63/2009_H1N1,
A/Boston/65/2009_H1N1,
A/Boston/71/2009_H1N1,
A/Boston/84/2009_H1N1,
A/Boston/86/2009_H1N1,
A/Boston/92/2009_H1N1,
A/Boston/93/2009_H1N1,
A/Boston/2/2009_H1N1,
A/Boston/6/2009_H1N1,
A/Boston/10/2009_H1N1,
A/Boston/22/2009_H1N1,
A/Boston/23/2009_H1N1,
A/Boston/25/2009_H1N1,
A/Boston/54/2009_H1N1,
A/Boston/59/2009_H1N1,
A/Boston/61/2009_H1N1,
A/Boston/70/2009_H1N1,
A/Boston/74/2009_H1N1,
A/Boston/77/2009_H1N1,
A/Boston/78/2009_H1N1,
A/Boston/81/2009_H1N1,
A/Boston/94/2009_H1N1,
A/Boston/32/2009_H1N1,
A/Boston/79/2009_H1N1,
A/California/VRDL372/2009_H1N1,
A/California/VRDL190/2009_H1N1,
A/California/VRDL191/2009_H1N1,
A/Boston/47/2009_H1N1,
A/HongKong/H090_682_V1_0_/2009_H1N1,
A/HongKong/H090_718_V1_0_/2009_H1N1,
A/HongKong/H090_744_V1_0_/2009_H1N1,
A/NewYork/08_1326/2008_H1N1,
A/Thailand/Siriraj_3043/2009_H1N1,
A/Japan/WRAIR1226P/2009_H1N1,
A/SouthKorea/WRAIR1238P/2009_H1N1,
A/DaNang/DN302/2008_H1N1,
A/HaNoi/BT241/2008_H1N1,
A/DaNang/DN345/2008_H1N1,

A/Hue/H257/2008_H1N1,

A/Hue/H274/2008_H1N1,

A/Hue/H339/2008_H1N1,

A/Hue/H386/2008_H1N1,

A/Hue/H396/2008_H1N1,

A/Hue/H413/2008_H1N1,

A/Hue/H423/2008_H1N1,

A/Hue/H432/2008_H1N1,

A/KhanhHoa/KH161/2008_H1N1,
A/KhanhHoa/KH57/2008_H1N1,
A/HaNoi/N229/2008_H1N1,
A/HaNoi/TX200/2008_H1N1,
A/HaNoi/TX233/2008_H1N1,
A/TayNinh/HCM1329/2008_H1N1,
A/Boston/80/2009_H1N1,
A/Boston/100/2009_H1N1,
A/Malaysia/1999752/2008_H1N1,
A/Malaysia/2155235/2009_H1N1,
A/Malaysia/2143035/2009_H1N1,
A/Malaysia/2104192/2008_H1N1,
A/Malaysia/2156486/2009_H1N1,
A/Kentucky/04/2009_H1N1,
A/Indiana/08/2009_H1N1,
A/Michigan/14/2009_H1N1,
A/Michigan/31/2009_H1N1,
A/HongKong/H090_766_V1_0_/2009_H1N1,
A/Taiwan/2832/2008_H1N1,
A/Naypyitaw/M499/2008_H1N1,
A/Cambodia/21/2007_H1N1,
A/Naypyitaw/M783/2008_H1N1,
A/Gansu/Chenguan/1129/2007_H1N1,
A/Yokohama/91/2007_H1N1,
A/Kansas/UR06_0104/2007_H1N1,
A/Massachusetts/05/2007_H1N1,
A/Minnesota/23/2007_H1N1,
A/Texas/31/2007_H1N1,
A/Mississippi/03/2001_H1N1,
A/England/494/2006_H1N1,
A/Georgia/20/2006_H1N1,
A/duck/InteriorAlaska/7MP1582/2007_H1N1

Supplementary figures

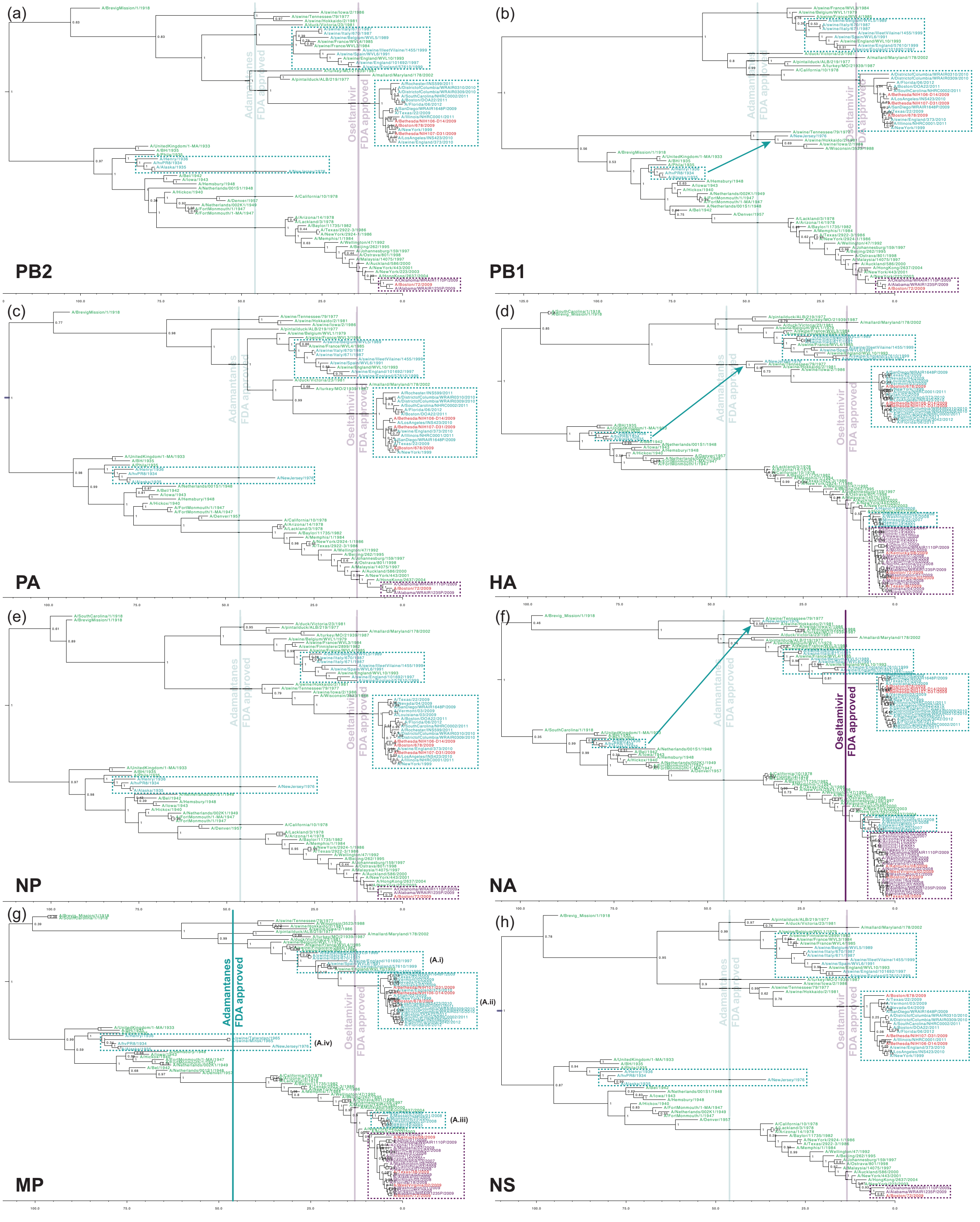

Figure S1 (Previous page). Dated phylogenies of drug-resistant Influenza A/H1N1 gene segments. Panels (a)-(h) represent inference for each of the eight segments. Green sequence labels indicate an absence of Adamantane or Oseltamivir resistance, blue the presence of Adamantane resistance, purple the presence of Oseltamivir resistance, and red both Adamantane and Oseltamivir resistance. The horizontal axis represents time in years since 2012 while the vertical bars show FDA approval for Adamantane (blue; 1966) and Oseltamivir (purple; 1999); colors of these bars are shaded for gene segments not involved in a particular resistance. Dotted boxes indicate resistance clades and main reassortment events. Arrows in panels (b), (d) and (f) represent reassortment of a particular gene segment of A/New Jersey/1976 between North American and Eurasian swine clades. Node labels represent posterior probabilities.

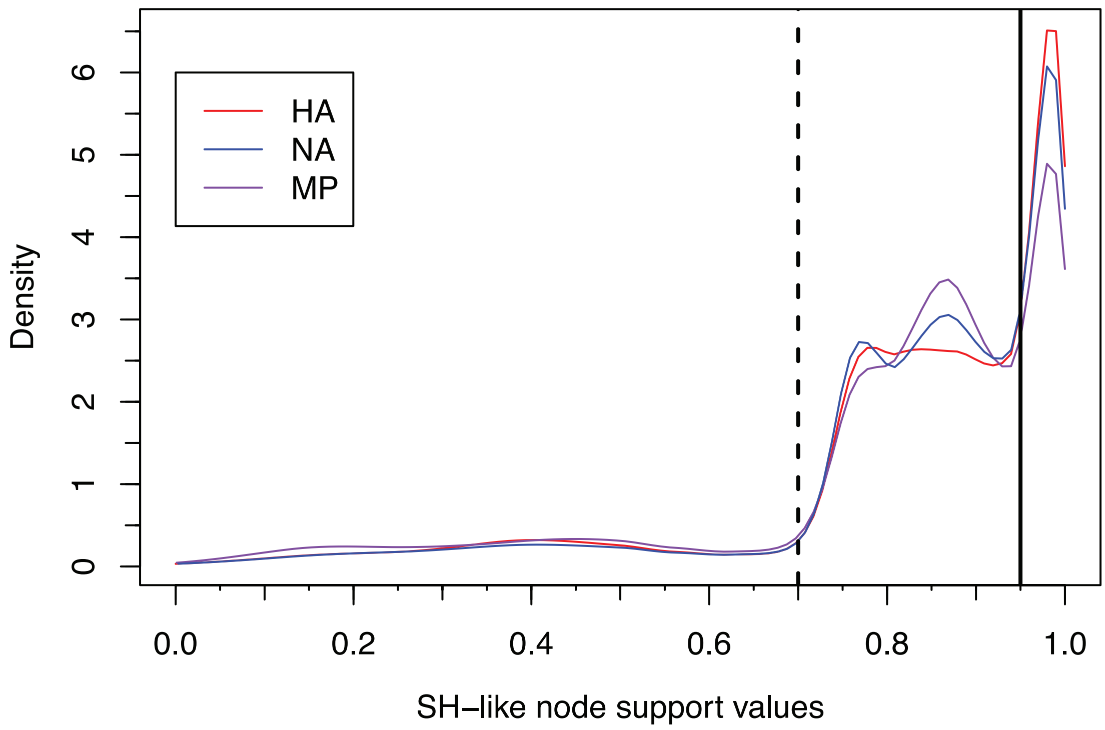

Figure S2. Distribution of node support values in the exhaustive phylogenetic analyses. Support values were computed as the SH-like approximate likelihood ratio test *P*-values (Methods) for the HA tree (red; 24,168 sequences), the NA tree (blue; 20,888 sequences) and the MP tree (purple; 19,932 sequences). Only nonzero support values were considered. Vertical black bars represent the 70% (broken line) and 95% (solid line) thresholds.
